# Thromboprophylaxis patterns and determinants in critically ill patients: a multicenter audit

**DOI:** 10.1186/cc13844

**Published:** 2014-04-25

**Authors:** François Lauzier, John Muscedere, Éric Deland, Demetrios Jim Kutsogiannis, Michael Jacka, Diane Heels-Ansdell, Mark Crowther, Rodrigo Cartin-Ceba, Michael J Cox, Nicole Zytaruk, Denise Foster, Tasnim Sinuff, France Clarke, Patrica Thompson, Steven Hanna, Deborah Cook

**Affiliations:** 1 Centre de recherche du CHU de Québec-Axe Santé des populations et pratiques optimales en santé, Traumatologie-urgence-soins intensifs, Division de soins intensifs adultes, départements de médecine et d'anesthésiologie, Université Laval, 1401 18e Rue, Québec, Québec, Canada G1J 1Z4; 2 Department of Medicine, Queen’s University, 94 Stuart Street, Kingston, Ontario, Canada K7L 3N6; 3 Département de Médecine, Université de Sherbrooke, 3011 12e avenue Nord, Sherbrooke, Québec, Canada J1H 5N4; 4 Division of Critical Care Medicine, Faculty of Medicine and Dentistry, University of Alberta, 2-124 Clinical Sciences Building, 8440-112 Street, Edmonton, Alberta, Canada T6G 2B7; 5 Department of Clinical Epidemiology and Biostatistics, McMaster University, 1200 Main Street West, Hamilton, Ontario, Canada L8N 3Z5; 6 Department of Medicine, McMaster University, 1200 Main Street West, Hamilton, Ontario, Canada L8N 3Z5; 7 Division of Pulmonary and Critical Care Medicine, Mayo Clinic, 200 First Street SW, Rochester MN 55905, USA; 8 Department of Pulmonary and Critical Care, St John’s Mercy Hospital, 3437 Caroline Mall, St. Louis MO 63104-1111, USA; 9 Division of Critical Care, Vancouver General Hospital, 855 West 12th Ave, Vancouver, British Columbia, Canada V5Z 1M9; 10 Department of Critical Care Medicine and Sunnybrook Research Institute, Sunnybrook Health Sciences Center, 2075 Bayview Avenue, Room D1.08, Toronto, Ontario, Canada M4N 3M5; 11 Interdepartmental Division of Critical Care, University of Toronto, R. Fraser Elliott Building, 190 Elizabeth St. Suite 3-805, Toronto, Ontario, Canada M5G 2C4

## Abstract

**Introduction:**

Heparin is safe and prevents venous thromboembolism in critical illness. We aimed to determine the guideline concordance for thromboprophylaxis in critically ill patients and its predictors, and to analyze factors associated with the use of low molecular weight heparin (LMWH), as it may be associated with a lower risk of pulmonary embolism and heparin-induced thrombocytopenia without increasing the bleeding risk.

**Methods:**

We performed a retrospective audit in 28 North American intensive care units (ICUs), including all consecutive medical-surgical patients admitted in November 2011. We documented ICU thromboprophylaxis and reasons for omission. Guideline concordance was determined by adding days in which patients without contraindications received thromboprophylaxis to days in which patients with contraindications did not receive it, divided by the total number of patient-days. We used multilevel logistic regression including time-varying, center and patient-level covariates to determine the predictors of guideline concordance and use of LMWH.

**Results:**

We enrolled 1,935 patients (62.3 ± 16.7 years, Acute Physiology and Chronic Health Evaluation [APACHE] II score 19.1 ± 8.3). Patients received thromboprophylaxis with unfractionated heparin (UFH) (54.0%) or LMWH (27.6%). Guideline concordance occurred for 95.5% patient-days and was more likely in patients who were sicker (odds ratio (OR) 1.49, 95% confidence interval (CI) 1.17, 1.75 per 10-point increase in APACHE II), heavier (OR 1.32, 95% CI 1.05, 1.65 per 10-m/kg^2^ increase in body mass index), had cancer (OR 3.22, 95% CI 1.81, 5.72), previous venous thromboembolism (OR 3.94, 95% CI 1.46,10.66), and received mechanical ventilation (OR 1.83, 95% CI 1.32,2.52). Reasons for not receiving thromboprophylaxis were high risk of bleeding (44.5%), current bleeding (16.3%), no reason (12.9%), recent or upcoming invasive procedure (10.2%), nighttime admission or discharge (9.7%), and life-support limitation (6.9%). LMWH was less often administered to sicker patients (OR 0.65, 95% CI 0.48, 0.89 per 10-point increase in APACHE II), surgical patients (OR 0.41, 95% CI 0.24, 0.72), those receiving vasoactive drugs (OR 0.47, 95% CI 0.35, 0.64) or renal replacement therapy (OR 0.10, 95% CI 0.05, 0.23).

**Conclusions:**

Guideline concordance for thromboprophylaxis was high, but LMWH was less commonly used, especially in patients who were sicker, had surgery, or received vasopressors or renal replacement therapy, representing a potential quality improvement target.

## Introduction

Thromboprophylaxis is a key component of care for critically ill patients because of their high risk of venous thromboembolism [1] and because heparin is an effective and safe prevention strategy. The Stanford University Evidence Based Practice Center rates thromboprophylaxis as the foremost patient safety initiative for hospitalized patients [2]. Moreover, the Joint Commission now specifies thromboprophylaxis as a key quality measure for hospitalized patients [3] and thromboprophylaxis is also a hospital accreditation metric in Canada [4].

Analysis of a large registry of 175,665 critically ill adult patients in 134 ICUs in Australia and New Zealand from 2006 to 2010 showed a significant association between omission of early thromboprophylaxis and hospital mortality after adjusting for covariates, including multiple trauma, sepsis, cardiac arrest, and preexisting metastatic cancer [5]. From a patient and healthcare system perspective, ascertaining current practice and ensuring that it is commensurate with current best evidence is crucial. We therefore conducted a multicenter audit of thromboprophylaxis in medical–surgical critically ill patients to identify the types and rates of thromboprophylaxis and to analyze factors associated with appropriate use. We hypothesized that approximately 80% of patients would receive some anticoagulant, reflecting approximately 70% of eligible ICU-days, and that low molecular weight heparin (LMWH) would be used less than unfractionated heparin (UFH) [6].

## Materials and methods

### Design

We conducted a multicenter retrospective 1-month practice audit of all consecutive patients admitted to the ICU between 1 November and 30 November 2011 in 26 centers across Canada and two centers in the United States to record thromboprophylaxis practices. We excluded patients admitted for less than 12 hours and patients admitted directly from the operating or recovery room after a cardiac surgery or neurosurgical procedure.

### Pilot reliability study

Case report forms and an implementation manual were developed and pretested by two research coordinators, adapted from prior studies [7,8]. We conducted a structured, independent, duplicate chart abstraction exercise to identify points of data disagreement, to clarify methodology, and to enhance the efficiency and validity of the audit process. Two research coordinators from eight participating centers reviewed the case report forms and implementation manual, and then each independently audited five charts, abstracting 27 baseline demographic variables and 30 daily data variables for each patient’s length of ICU stay, which ranged from 2 to 60 days. Only 2% of variables were discordant overall. This calibration exercise mitigated discordance within and across centers, and improved the operational efficiency of the audit [9].

### Audit

Data were abstracted until death or ICU discharge, censored at 60 days. Trained research coordinators collected demographics and baseline characteristics (age, sex, Acute Physiology and Chronic Health Evaluation (APACHE) II score [10], medical vs. surgical status, ICU admitting diagnosis), and relevant clinical outcomes (deep vein thrombosis, pulmonary embolism, major bleeding [11], heparin-induced thrombocytopenia, mortality). Venous thromboembolism events were diagnosed by the treating physicians based on clinical judgment and objective testing.

Pharmacologic prophylaxis (UFH, LMWH, warfarin, danaparoid, other agents), mechanical prophylaxis (antiembolic stockings, pneumatic compression devices), therapeutic anticoagulation, antiplatelet treatments, and use of inferior vena cava filters were captured daily, as well as factors potentially modulating prescribing such as laboratory values (for example, platelet count), outcomes (for example, bleeding), confirmatory tests (for all venous thromboembolism events), and process of care variables (for example, mobility). We also recorded characteristics of participating centers, including the number of ICU beds, the presence of a dedicated thrombosis service, trauma service or ICU quality improvement team, and whether thromboprophylaxis was administered using preprinted orders or computerized physician order entry.

### Adjudication

Venous thromboembolism events and bleeding events were adjudicated by one investigator unaware of the use of thromboprophylaxis using established and validated classification systems. For venous thromboembolism events, recent trial definitions were used [12]. Bleeding was classified as major if it was life threatening due to hypovolemic shock (for example, ruptured abdominal aortic aneurysm) or at a critical site (for example, intracranial), if the bleeding was overtly clinically important and was associated with one of several criteria within 24 hours of the bleed (decrease in hemoglobin >20 g/l, transfusion ≥2 packed red blood cells, decrease in systolic blood pressure >20 mmHg, or increase in heart rate >20 bpm in the absence of other causes), or if the bleeding required an invasive intervention (for example, reoperation) [11,12]. Heparin-induced thrombocytopenia was diagnosed by serotonin release assay [13]. Thrombosis was attributed to heparin-induced thrombocytopenia if it occurred within 1 week of the positive serologic test.

### Analysis

We reported continuous data as the mean and standard deviation or the median and interquartile range when data were skewed. We reported absolute numbers of patients or days, and proportions. We used *t* tests and Wilcoxon rank-sum tests to compare continuous data and Fisher’s exact test to compare proportions.

We analyzed thromboprophylaxis overall and by center. Our primary outcome was guideline concordance with the 2008 American College of Chest Physicians Antithrombotic Therapy and Prevention of Thrombosis Guidelines’ 1A recommendation for daily heparin thromboprophylaxis (either UFH or LWMH) for all critically ill patients unless contraindications exist [14]. We calculated a guideline concordance rate for any type of heparin prophylaxis (UFH or LWMH) or therapeutic heparin, in those patients receiving it, by center and overall. Specifically, guideline concordance was defined as ICU days in which eligible patients for any type of pharmacologic thromboprophylaxis (any ICU patient without contraindications) were receiving it as recommended, plus noneligible patients who were not receiving it as recommended, divided by the total number of ICU patient-days. By eligible patients, we refer to those being in the ICU with no contraindications to pharmacologic prophylaxis (for example, active bleeding, high risk of bleeding, suspected or proven heparin-induced thrombocytopenia, or imminent or recent invasive procedure within 24 hours). Other reasons or no clear reasons were not considered contraindications.

To analyze the factors associated with guideline concordance, we used multilevel logistic regression, analyzing repeated measurements of concordance within patients and within centers. Possible determinants included two factors at the level of center (dedicated thrombosis service, use of preprinted orders), five factors at the level of patients (medical versus surgical admission, APACHE II score, cancer, history of venous thromboembolism events, or body mass index), and three time-varying factors at the level of patient-days (invasive mechanical ventilation, inotropes or vasopressors, and renal replacement therapy). Patient factors were therefore measured at either baseline (for example, cancer) or on a daily basis (for example, renal replacement therapy). We calculated odds ratios (ORs) and 95% confidence intervals (CIs). We considered factors significant at the *P* < 0.05 level.

In a second regression analysis, we examined factors associated with LMWH thromboprophylaxis rather than UFH thromboprophylaxis, including only those patient-days on which the patient received doses of either agent. This was based on a recent systematic review of randomized trials of LMWH versus UFH in medical–surgical patients performed by our group [15]. We considered the same covariates as in the first regression.

### Research ethics

This retrospective audit was reviewed and approved by each participating center’s Research Ethics Board (see Acknowledgements), waiving the need for informed consent.

## Results

We enrolled patients from 26 Canadian centers and two US centers. The centers contributed a median (interquartile range) of 55.5 (42.5, 74.0) patients to the audit. Participating centers had a mean (standard deviation) of 22.6 (9.8) ICU beds. Among the 28 centers, a dedicated thrombosis service existed in nine centers (32.1%), a dedicated trauma service in 17 centers (60.7%), and a dedicated ICU quality improvement team in 19 centers (67.9%). Thromboprophylaxis prescribing was facilitated by preprinted orders in 21 centers (75.0%), and by computerized physician order entry in six centers (21.4%).

We included 1,935 patients (mean age 62.3 ± 16.7) with a mean APACHE II score of 19.1 ± 8.3. Baseline characteristics are shown in Table 1 and patient outcomes in Table 2. Venous thromboembolic events were uncommon: leg thrombi (42 patients, 2.2%), nonleg thrombi (52 patients, 2.7%), and pulmonary embolism (36 patients, 1.9%). Heparin-induced thrombocytopenia occurred in two patients (0.001%), associated with venous thromboembolic events in both. Major bleeding occurred in 187 patients (9.7%). Among these patients, 74 were receiving either LMWH or UFH for thromboprophylaxis on their first day of bleeding. Mortality was 12.5% (242 patients) in the ICU and 19.4% (375 patients) in hospital.

**Table 1:** Baseline patient characteristics

**Characteristic**	**All patients (*n*** **= 1,935)**
Age (years)	62.3 (16.7)
APACHE II score	19.1 (8.3)
Females	869 (44.9)
Admission diagnosis	
Cardiovascular	283 (14.6)
Respiratory	517 (26.7)
Gastrointestinal	313 (16.2)
Renal	53 (2.7)
Neurologic	215 (11.1)
Sepsis	240 (12.4)
Trauma	14 (0.7)
Metabolic	133 (6.9)
Hematologic	16 (0.8)
Other medical	71 (3.7)
Other surgical	80 (4.1)
Location prior to ICU	
Operating room/recovery room	485 (25.1)
Emergency room	669 (34.6)
Ward	480 (24.8)
Other hospital ICU	83 (4.3)
Other hospital ward	218 (11.3)
Medical admission	1453 (75.1)
Mechanical ventilation	
Invasive	997 (51.5)
Non-invasive only	132 (6.8)
None	806 (41.6)
Vasopressor/inotropes	611 (31.6)
Dialysis	67 (3.5)

Data presented as mean (standard deviation) or number (percentage). Characteristics of the 1,935 patients included in this audit on the first day of ICU admission, from 28 participating ICUs. APACHE, Acute Physiology and Chronic Health Evaluation.

**Table 2:** Patient outcomes

**Outcome**	**All patients (*n*** **= 1,935)**
Major outcomes	
ICU mortality	242 (12.5)
Hospital mortality	375 (19.4)
Re-admitted to ICU	55 (2.8)
ICU length of stay (days)	4 (2 to 7)
Hospital length of stay (days)	12 (6 to 24)
Adjudicated outcomes	
Leg thrombus	42 (2.2)
Nonleg thrombus^a^	52 (2.7)
Pulmonary embolism	36 (1.9)
Any venous thromboembolism	117 (6.0)
Heparin-induced thrombocytopenia	3 (0.2)
Major bleeding	187 (9.7)
Any bleeding	457 (23.6)

Data presented as number (percentage) or median (interquartile range). Venous thromboembolic outcomes, length of stay and mortality status of the 1,935 patients included in this audit, from 28 participating ICUs. Only the first ICU admissions during the audit month were considered. Outcomes are not mutually exclusive. ^a^Thromboses occurring in sites other than the lower extremities, including the head and neck, trunk, and upper extremities.

**Figure 1:**
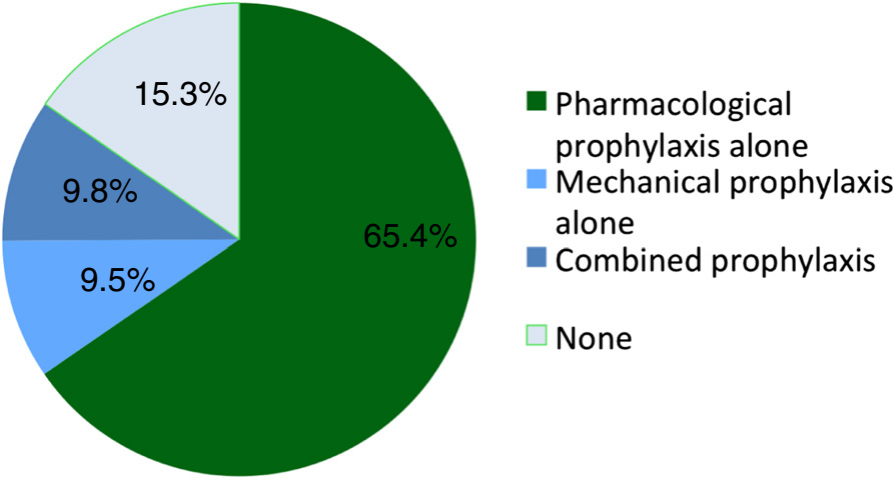
**Thromboprophylaxis strategy in medical–surgical patients.** Proportions of patient-days for each thromboprophylaxis strategy used (pharmacological, mechanical, combined, none).

Overall, 1,619 patients (83.7%) received some form of anticoagulant during their ICU stay. Pharmacologic thromboprophylaxis was with UFH in 1,044 patients (54.0%) or with LMWH in 535 patients (27.6%), whereas 390 patients (20.2%) were therapeutically anticoagulated at some time with UFH, warfarin, LMWH, or danaparoid for venous thromboembolism or other indication such as atrial fibrillation and acute coronary syndrome (Table 3). When considering patient-days as the unit of analysis, prophylaxis patterns were similar. Pharmacologic prophylaxis was administered for 65.4% of patient-days. There were 1,957 of 12,756 patient-days (15.3%) during which no thromboprophylaxis (neither pharmacologic nor mechanical) was administered (Figure 1).

**Table 3:** Use of anticoagulants

	**Patients (*n*** **= 1,935)**	**Patient-days (*n*** **= 12,756)**
Prophylactic anticoagulants		
Subcutaneous LMWH	535 (27.6)	2,687 (21.1)
Subcutaneous UFH^a^	1,044 (54.0)	5,504 (43.1)
Therapeutic anticoagulants		
Any therapeutic anticoagulant	390 (20.2)	1,693 (13.3)
Intravenous UFH	284 (14.7)	1,210 (9.5)
LMWH	35 (1.8)	119 (0.9)
Coumadin	94 (4.9)	324 (2.5)
Danaparoid	5 (0.3)	7 (0.05)
Other^b^	52 (2.7)	168 (1.3)
Any of the above	1,619 (83.7)	9,589 (75.2)

Anticoagulation management for the 1,935 patients included in this audit, and ICU patient-days. Some patients received more than one type of anticoagulation during their ICU stay. LMWH, low molecular weight heparin; UFH, unfractionated heparin. ^a^Refers to doses of UFH that were not ordered to target an activated partial thromboplastin time. ^b^Thrombolytic agents (streptokinase, recombinant tissue plasminogen activator), argatroban, fondaparinux, eptifibatide, dabigatran, rivaroxaban.

We documented guideline concordance for 12,186/12,756 (95.5%) patient-days. The range of guideline concordance in participating centers ranged from 81.3 to 100.0%. The highest level of patient activity during these 570 patient-days of nonconcordance included bed rest (363 patient-days, 64.0%), transferring to a chair (100 patient-days, 17.5%), and walking (105 patient-days, 18.4%), with data missing for 2 patient-days. The respiratory status during nonconcordant patient-days was spontaneously breathing (331 patient-days, 58.1%), non-invasive ventilation (215 patient-days, 37.7%), and invasive mechanical ventilation (24 patient-days, 4.2%). We did not identify any patients who received heparin when it was contraindicated.

Factors associated with guideline concordance with thromboprophylaxis are reported in Table 4. Guideline concordance was more likely in patients who were sicker (OR = 1.49, 95% CI = 1.17, 1.75 for each 10-point increase in APACHE II score), in patients who were heavier (OR = 1.32, 95% CI = 1.05, 1.65 for each 10-point increase in body mass index), in patients with cancer (OR = 3.22, 95% CI = 1.81, 5.72), in patients with a history of venous thromboembolism (OR = 3.94, 95% CI = 1.46, 10.66), and among those receiving mechanical ventilation (OR = 1.83, 95% CI = 1.32, 2.52).

**Table 4:** Factors associated with guideline concordance: multilevel logistic regression

**Three-level model**	**Odds ratio (95% CI)**	***P *** **value**
Patient factors		
Surgical admission	1.09 (0.68, 1.75)	0.718
APACHE II score (10-point increase)	1.49 (1.17, 1.89)	0.001
Cancer^a^	3.22 (1.81, 5.72)	<0.001
History of venous thromboembolism	3.94 (1.46, 10.66)	0.007
Body mass index (10-point increase)	1.32 (1.05, 1.65)	0.018
Daily factors^b^		
Any dialysis	0.79 (0.45, 1.40)	0.422
Invasive mechanical ventilation	1.83 (1.32, 2.52)	<0.001
Vasopressors or inotropes	1.26 (0.90, 1.78)	0.184
Site factors		
Dedicated thrombosis consulting service	1.91 (0.95, 3.86)	0.069
Preprinted orders including thromboprophylaxis	1.00 (0.51, 1.98)	0.989

Results of the multilevel logistic regression model examining determinants of guideline concordance (use of any pharmacological thromboprophylaxis unless contraindications exist). The three levels are center, patient, and ICU-day. There were 10,540 patient-days (10,154 with concordance, 386 without), *n* = 1,533 patients. Body mass index values were missing for 402 of the 1,935 patients enrolled in the study. These patients were therefore excluded from the regression analysis. APACHE, Acute Physiology and Chronic Health Evaluation; CI, confidence interval. ^a^Cancer refers to lymphoma, metastatic cancer, leukemia, multiple myeloma, active solid malignancy, or history of solid malignancy. ^b^Daily factors reflect exposure in the preceding 3 days.

For 3,167 patient-days (24.8%) where no form of anticoagulant was administered, the reasons given were high risk of bleeding (44.5%), bleeding (16.3%), no reason evident (12.9%), invasive procedure (10.2%), nighttime admission to or discharge from the ICU (9.7%), life-support limitation (6.9%), perception that it was unnecessary (4.8%), other (2.0%), or suspected or proven heparin-induced thrombocytopenia (1.4%) (Table 5).

**Table 5:** Reason for not using anticoagulant

**Reason**	**3,167 patient-days with no anticoagulant**
High risk of bleeding	1,410 (44.5)
Bleeding	517 (16.3)
Invasive procedure/surgery	324 (10.2)
Nighttime admission or discharge	306 (9.7)
Limiting life support	218 (6.9)
Perceived unnecessary	153 (4.8)
Other^a^	62 (2.0)
Suspected/proven heparin-induced thrombocytopenia	45 (1.4)
No reason evident	408 (12.9)

Reasons for no anticoagulation for 3,167 patient-days among 1,116 patients. ^a^Examples include severe anemia, mildly abnormal laboratory values, prescribing omission, pharmacy error, expected short-term ICU admission, ambulation, and patient declined.

Factors associated with prescription of LMWH instead of UFH per patient-day are reported in Table 6. LMWH was less likely used than UFH in sicker patients (OR = 0.65, 95% CI = 0.48, 0.89 for each 10-point increase in APACHE II score), in surgical patients versus medical patients (OR = 0.41, 95% CI = 0.24, 0.72), in those receiving inotropes or vasopressors (OR = 0.47, 95% CI = 0.35, 0.64), and in patients receiving renal replacement therapy (OR = 0.10, 95% CI = 0.05, 0.23).

**Table 6:** Factors associated with LMWH rather than UFH thromboprophylaxis: multilevel logistic regression

**Three-level model**	**Odds ratio (95% CI)**	***P *** **value**
Patient factors		
Surgical admission	0.41 (0.24, 0.72)	0.002
APACHE II score (10-point increase)	0.65 (0.48, 0.89)	0.007
Cancer^a^	1.12 (0.64, 1.94)	0.692
History of venous thromboembolism	1.18 (0.50, 2.76)	0.704
Body mass index (10-point increase)	1.12 (0.88, 1.44)	0.362
Daily factors^b^		
Any dialysis	0.10 (0.05, 0.23)	<0.001
Invasive mechanical ventilation	0.77 (0.56, 1.06)	0.105
Vasopressors or inotropes	0.47 (0.35, 0.64)	<0.001
Site factors		
Dedicated thrombosis consulting service	4.20 (0.62, 28.60)	0.135
Preprinted orders including thromboprophylaxis	1.79 (0.24, 13.44)	0.556

Results of the multilevel logistic regression model examining determinants of LMWH rather than UFH thromboprophylaxis. The three levels are center, patient, and ICU-day. There were 6,856 patient-days (2,182 with LMWH prophylaxis), *n* = 1,181 patients. Body mass index values were missing for 402 of the 1,583 patients who received thromboprophylaxis. These patients were therefore excluded from the regression analysis. APACHE, Acute Physiology and Chronic Health Evaluation; CI, confidence interval; LMWH, low molecular weight heparin; UFH, unfractionated heparin. ^a^Cancer refers to lymphoma, metastatic cancer, leukemia, multiple myeloma, active malignancy, or history of malignancy. ^b^Daily factors reflect exposure in the preceding 3 days.

**Figure 2:**
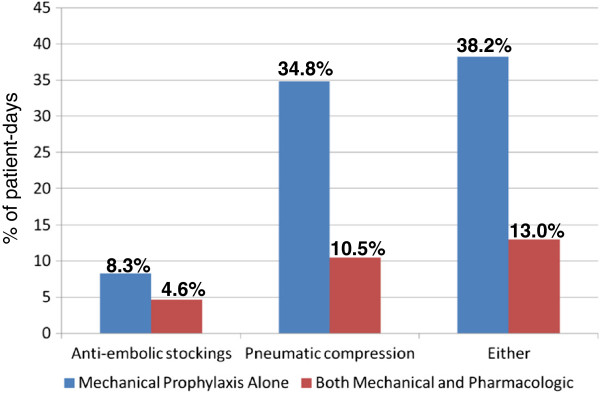
**Mechanical prophylaxis according to concomitant pharmacologic thromboprophylaxis.** Proportions of patient-days for each type of mechanical thromboprophylaxis used (anti-embolic stockings, pneumatic compression, either type) depending on the use of concomitant pharmacological thromboprophylaxis.

Mechanical prophylaxis was ordered less often than pharmacologic prophylaxis (5.5% patient-days for anti-embolic stockings and 16.5% patient-days for pneumatic compression devices). These two devices were most often ordered when no anticoagulant was administered (Figure 2). More specifically, anti-embolic stockings were administered for 8.3% patient-days, and pneumatic compression devices on 34.8% patient-days. Overall, there were 1,245 patient-days (9.8%) during which both mechanical prophylaxis (anti-embolic stockings and pneumatic compression devices) and pharmacological prophylaxis were applied. Inferior vena cava filters were inserted in 31 patients (1.6%), for a total of 157 patient-days. Two-thirds of inferior vena cava filters were inserted prophylactically (21/31 filters, 67.7%), and two patients developed a leg deep venous thrombosis after the filter insertion during their ICU stay.

## Discussion

In this 1-month multicenter audit, we observed a guideline concordance for pharmacological thromboprophylaxis for 95.5% of ICU-days in medical–surgical critically ill patients. Guideline concordance for pharmacological thromboprophylaxis was more likely in sicker and heavier patients, and in patients with cancer, with a history of venous thromboembolism, and those receiving mechanical ventilation. LMWH was less commonly used than UFH, especially in patients who were sicker, who had surgery, or who received vasoactive drugs or renal replacement therapy.

The guideline concordance documented in this audit was somewhat higher than previous audits [7,16]. This increased concordance may reflect the growing number of randomized trials supporting the use of heparin in various populations, including in the ICU. Time has allowed for the passive diffusion of evidence into practice, and generalized application of heparin thromboprophylaxis. The encoding of thromboprophylaxis into hospital accreditation may also play a role. Our findings may reflect the low cost of heparin relative to other preventive or therapeutic interventions used in the ICU. High guideline concordance of pharmacologic thromboprophylaxis as a relatively simple intervention contrasts with some other multifaceted quality improvement initiatives such as ventilator-associated pneumonia prevention [17] for which there are several components (for example, body position, mechanical interventions, pharmacologic approaches).

Use of pharmacologic thromboprophylaxis was significantly more likely in patients with high illness severity, a diagnosis of cancer, a history of venous thromboembolism event, and a high body mass index. Clinician awareness of risk factors may have driven the higher penetrance of pharmacologic thromboprophylaxis use for patients with these characteristics. Prior critical care research has shown that the risk of a venous thromboembolism event is greater in patients with a high APACHE II score [18], cancer [19], personal or family history of venous thromboembolism [20,21], and greater weight [22-24]. Inadequate dosing in obese patients leading to lower anti-Xa levels [25] may explain this association [26,27]. Of the three advanced life supports examined, only mechanical ventilation was significantly associated with guideline concordance, possibly reflecting perceived higher risk of a venous thromboembolism event in mechanically ventilated patients [28,29]. In terms of center effects, neither the presence of a dedicated thrombosis consulting service nor the use of preprinted orders was associated with guideline concordance, adjusting for other patient-specific factors. Although drug-prescribing modification is amenable to preprinted orders, the impact has not been well studied in the ICU.

As hypothesized, LMWH was administered less often than UFH, which is consistent with a national Austrian audit of 325 critically ill patients documenting lower use of LMWH [30]. In early 2013, the Surviving Sepsis Campaign issued a 1B recommendation to use LMWH daily thromboprophylaxis instead of UFH twice-daily thromboprophylaxis in the absence of contraindications [31]. This recommendation was partly based on the multinational Prophylaxis for Thromboembolism in Critical Care Trial (PROTECT) in 3,764 critically ill patients showing that dalteparin significantly reduced the risk of pulmonary embolism in critically ill patients compared with UFH, with no difference in major bleeding and a trend toward lower rates of deep vein thrombosis, overall venous thromboembolism events, and heparin-induced thrombocytopenia [12]. Subsequently, a recent meta-analysis of five randomized trials enrolling more than 5,000 medical–surgical critically ill patients showed that LMWH reduced rates of overall and symptomatic pulmonary embolism compared with UFH, but not overall and symptomatic deep venous thrombosis or mortality, while major bleeding was not different [15]. The gap in care regarding the use of LMWH is moderately large, and may represent a quality improvement target. A prospective economic evaluation conducted alongside the PROTECT study indicated that a strategy of thromboprophylaxis was the least costly strategy until the cost of dalteparin rose from a base case cost of $8.13 to $183 per dose (R Fowler *et al.*, Cost-effectiveness of dalteparin versus unfractionated heparin for the prevention of venous thromboembolism in critically ill patients: a prospective comparative economic evaluation of the Prophylaxis for Thromboembolism in Critical Care Trial (PROTECT), submitted). There was no threshold in which lowering the acquisition cost of UFH favored this prophylaxis strategy.

In our study, patients receiving inotropes or vasopressors were 50% less likely to receive LMWH than UFH. Such patients are at higher risk of venous thromboembolism [20], possibly due to the concomitant proinflammatory and procoagulant state, or decreased subcutaneous heparin bioavailability as suggested by lower anti-Xa levels [32,33] related to peripheral blood shunting or edema [34]. LMWH may be less likely prescribed to patients receiving inotropes or vasopressors due to fear of bleeding, as these patients have lower platelet counts, higher International Normalized Ratio values, and higher partial thromboplastin time values than those not receiving inotropes or vasopressors. Surgical patients were also less likely to receive LMWH than UFH compared with medical patients, which may reflect concern about increased risk of postoperative bleeding. This situation is paradoxical in that the relative benefit of LMWH over UFH is stronger in surgical populations [35] than in medical populations [1]. Patients receiving renal replacement therapy were also significantly less likely to receive LMWH than UFH. This could reflect concern about LMWH bioaccumulation in renal insufficiency. However, dalteparin 5,000 U subcutaneously does not bioaccumulate, as demonstrated by undetectable mean anti-Xa levels in a multicenter study of ICU patients with a range of renal dysfunction including anuric renal failure [36]. Similarly, when administered at prophylactic doses to patients with a range of renal function, prophylactic tinzaparin did not bioaccumulate whereas enoxaparin did [37].

Mechanical thromboprophylaxis with either anti-embolic stockings or pneumatic compression devices was infrequent. Mechanical thromboprophylaxis was primarily used in patients who were currently bleeding or at risk of bleeding, which is congruent with the American College of Chest Physicians 2012 recommendation to use mechanical thromboprophylaxis for patients with contraindications to heparin [1], and the Grade 2C Surviving Sepsis Campaign recommendation [31]. Our observation that 6% and 17% of patient-days involved anti-embolic stockings and pneumatic compression devices, respectively, highlights the frequency of contraindications to pharmacological prophylaxis in medical–surgical patients. This observation also underscores the need for higher quality research on the effectiveness of mechanical prophylaxis, given the sparse data supporting their efficacy in this population [38]. Despite recommendations against the use of inferior vena cava filters for venous thromboembolism events and prophylaxis [14,36,39] and clear evidence that they cause thrombosis, the filters continue to be widely used for prevention. Although we did not examine removal rates in this audit, it is also concerning in real-world practice that less than 20% of retrievable filters are actually removed [40].

This study has several limitations. We could not incorporate physician factors as determinants of documented prophylaxis because physicians are numerous in the ICU on any given day (for example, attending, fellow, resident) and prescribers change often throughout a patient’s ICU stay, precluding the attribution of drug prescribing to one physician on any given day or for any given patient. Given the retrospective design, we could not concurrently survey clinicians to determine the rationale for their prescribing choices. We did not collect data after ICU discharge. In one observational study, survivors with resolving critical illness were less likely to receive thromboprophylaxis on the ward compared with within the ICU [29]. Finally, our collaboration with North American centers could to some extent explain why prophylactic UFH was preferentially used over LMWH, because LMWH was adopted sooner in Europe [41].

This study has several strengths. By building on our recent research to document [7,8], understand [42], implement [43], and test [12,37] thromboprophylaxis in the ICU, we examined whether and how clinicians use heparin thromboprophylaxis in this audit. There were several features of this study that contributed to its success – related to the project itself (relevant topic, simple design, manageable amount of data), the operations (a supportive methods center, user-friendly tools, formal training, provision of results to participating centers, funding), and the centers (commitment, skilled personnel, membership in a network in which the audit is embedded) [44]. Furthermore, we provided each participating hospital with patient-centered, site-specific data formulated as a quality improvement metric of guideline concordance designed for heparin thromboprophylaxis. The metric reflected individualized pharmacotherapeutic care and incorporated potentially changing daily thrombotic and bleeding risks over the ICU stay relevant to a broad case-mix of medical–surgical patients.

We included a large number of centers in North America enrolling a wide range of patients. By examining the largest and most heterogeneous group of medical–surgical ICU patients to date, we enhanced the generalizability of our findings. We conducted a pilot reliability study demonstrating perfect agreement on 98% of collected variables between two data abstractors, suggesting reliable data collection [9]. The comprehensive data collection included baseline premorbid conditions, and daily events and exposures over the ICU stay that influence thromboprophylaxis prescribing. We calculated concordance, which takes into account those who should and should not receive prophylaxis. We used ICU patient-days as the unit of analysis for guideline concordance because this acknowledges daily changes in a patient’s condition and drug prescribing, rather than treating each patient as concordant or not based on a threshold of concordance days [45]. We used multilevel modeling, which allows concurrent analysis of center and patient factors (fixed baseline characteristics and variable patient-days) as determinants of administration, and avoided overfitting [46].

## Conclusions

In summary, in this 1-month multicenter audit of thromboprophylaxis administration in a large cohort of medical–surgical critically ill patients, we documented widespread use of anticoagulation in prophylactic or therapeutic doses, greater use of UFH than LMWH, and mechanical prophylaxis primarily in patients who are bleeding or at risk of bleeding. Guideline concordance with any type of anticoagulant was high (95.5% per ICU patient-day) and reasons for noncompliance were poorly documented. Patients who were sicker, heavier, have cancer, or have prior VTE were more likely to receive pharmacological thromboprophylaxis. Patients who were sicker, who had surgery, or who received inotropes, vasopressors or renal replacement therapy were less likely to receive LMWH than UFH, representing a potential quality improvement target.

## Key messages

• UFH is more commonly used than LMWH for thromboprophylaxis in medical–surgical critically ill patients.

• Guideline concordance for pharmacological thromboprophylaxis of any type is 95.5% per ICU patient-day.

• Patients who were sicker, patients who were heavier, and patients with cancer or prior thrombotic events were more likely to receive pharmacological thromboprophylaxis.

• Patients who were sicker, who had surgery, or who received inotropes, vasopressors or renal replacement therapy were less likely to receive LMWH for thromboprophylaxis.

## Abbreviations

APACHE: Acute Physiology and Chronic Health Evaluation; CI: confidence interval; LMWH: low molecular weight heparin; OR: odds ratio; PROTECT: Prophylaxis for Thromboembolism in Critical Care Trial; UFH: unfractionated heparin.

## Competing interests

MC sat on advisory boards for Leo Pharma, Pfizer, Bayer, Boehringer Ingelheim, Alexion, CSL Behring, and Artisan Pharma; prepared educational materials for Pfizer, Octapharm, and CSL Behring; and provided expert testimony for Bayer. MC’s institution has received funding for research projects from Boehringer Ingelheim, Octapharm, Pfizer, and Leo Pharma. DC received peer review funding from CIHR for the randomized trial PROTECT comparing unfractionated heparin versus the low molecular weight dalteparin. Pfizer and Eisai Inc. donated dalteparin. NZ and DH-A received conference support from Pfizer. The remaining authors declare that they have no competing interests.

## Authors’ contributions

FL, JM, DJK, DH-A, MC, NZ, DF, SH, and DC contributed to study design. FL, JM, ED, RC-C, MJC, NZ, DF, FC, PT, TS, and DC participated in data collection. FL, DH-A, SH, and DC performed data analysis. FL, JM, ED, DJK, MJ, MJC, TS, RC-C, MC, SH, and DC interpreted the data. FL, DH-A, and DC drafted the manuscript. All authors revised the manuscript for important intellectual content and approved the submitted version.

## Participating CONECCKT-T Centers and Research Teams

*Canadian Investigators*: Dr Deborah Cook, France Clarke, and Nicole Zytaruk (St Joseph’s Healthcare, Hamilton); Dr Rick Hall, Lisa Julien, and Michelle St. Laurent (Halifax Infirmary and Victoria Hospital, Capital Health QEII Health Science Center, Halifax); Dr Maureen Meade, Lori Hand, and France Clarke (Hamilton Health Sciences-Hamilton General Hospital, Hamilton); Dr Francois Lauzier, and Ann Laberge (CHU de Québec-Hôpital de l’Enfant-Jésus, Québec); Dr Lauralyn McIntyre, Dr Guiseppe Pagliarello, Irene Watpool, Tracy McArdle, Allyshia Van Tol, Nicole Zito, Amanda Van Beinum, Lana Antoinette Castellucci, and Shawna Reddie (Ottawa Hospital, General Hospital and Civic Hospital, Ottawa); Dr Yoanna Skrobik, Johanne Harvey, and Brigitte Beauregard (Hôpital Maisonneuve-Rosemont, Montreal); Dr Martin Albert, David Williamson, Guilaine Coralie (Hôpital du Sacré-Coeur de Montréal, Montréal); Dr Sangeeta Mehta, Maedean Brown, and Rottem Kuint (Mount Sinai Hospital, Toronto); Dr Robert Fowler, Dr Taz Sinuff, Nicole Marinoff, Ashwati Raghunath, and Alexandra Rousseau (Sunnybrook Health Sciences Centre, Toronto); Dr John Marshall, Orla Smith, Yoon Lee, Melissa Wang, Pragma Roy, and Raphael Faraj (St. Michael’s Hospital, Toronto); Dr John Muscedere, Susan Fleury, and Nicole Godfrey (Kingston General Hospital, Kingston); Dr Donald Griesdale, Denise Foster, and Susan Logie (Vancouver General Hospital, Vancouver); Dr Greg Martinka, and Denise Foster (Richmond Hospital, Richmond, BC); Dr Peter Dodek, Betty-Jean Ashley, Sabrina Anis, Kelsey Brewer, and Sarah Shepherd (St. Paul’s Hospital, Vancouver); Dr Francois Lellouche, and Marie-Claude Ferland (Institut universitaire de cardiologie et de pneumologie de Québec, Québec); Dr Tim Karachi, France Clarke, and Nicole Zytaruk (Hamilton Health Sciences-Juravinski Hospital, Hamilton); Dr Michael Jacka, Marleen Irwin, Shanice Hannigan, and Emily Gentles (University of Alberta, Edmonton); Dr Gordon Wood, Fiona Auld, and Leslie Atkins (Vancouver Island Health Authority, Vancouver); Dr Éric Deland, Dr François Lamontagne, Dr Marylise Parent, and Chantal Langevin (Centre hospitalier universitaire de Sherbrooke, Sherbrooke); Dr Bojan Paunovic, Nicole Marten, and Shauna Eggerton (St Boniface Hospital, Winnipeg); Dr D Jim Kutsogiannis, Patrica Thompson, Darlene Jossy, and Samara Bush (Royal Alexandra Hospital, Edmonton); Dr Hari Kumar, and Patricia Shea (Saint John’s Regional Hospital, St. John’s New Brunswick); and Dr Marios Roussos, Sandra Stoger, Cindy Cryderman, Kathleen Romano, Amanda Carli, and Deanna Hicklin (Thunder Bay Regional Health Center, Thunder Bay).

*US Investigators*: Dr Rodrigo Cartin-Ceba, Laurie Meade, Gregory Wilson, Tami Krpata, and Alberto Marquez (Mayo Clinic, Rochester, MN); and Dr Michael Cox, Jackie O’Brien, Catherine Krause, Margaret Cyton, Kimberly Fowler, and Catherine Hubert (St John’s Mercy Medical Center, St Louis, MO).

*CONECCKT-T Methods Center*: France Clarke, Ceilidh Barlow Cash, Katherine Krolicki, Diane Heels-Ansdell, Laurel Grainger, and Nicole Zytaruk (McMaster University and St Joseph’s Healthcare, Hamilton, Canada).

## References

[B1] Kahn SR Lim W Dunn AS Cushman M Dentali F Akl EA Cook DJ Balekian AA Klein RC Le H Schulman S Murad MH Prevention of VTE in nonsurgical patients: antithrombotic therapy and prevention of thrombosis, 9th ed: American College of Chest Physicians evidence-based clinical practice guidelines Chest 2012 141 e195S e226S 22315261 10.1378/chest.11-2296 PMC3278052

[B2] Prevention of Venous Thromboembolism after Injury. Evidence Report/Technology Assessment: Number 22 [http://archive.ahrq.gov/clinic/epcsums/vtsumm.htm] PMC4781614 11925968

[B3] Venous Thromboembolism [http://www.jointcommission.org/venous_thromboembolism/]

[B4] Required Organizational Practices 2012 [http://www.accreditation.ca/sites/default/files/rop-handbook-2014-en.pdf]

[B5] Ho KM Chavan S Pilcher D Omission of early thromboprophylaxis and mortality in critically ill patients: a multicenter registry study Chest 2011 140 1436 1446 10.1378/chest.11-1444 21940768

[B6] Cook D McMullin J Hodder R Heule M Pinilla J Dodek P Stewart T Canadian ICU Directors Group Prevention and diagnosis of venous thromboembolism in critically ill patients: a Canadian survey Crit Care 2001 5 336 342 10.1186/cc1066 11737922 PMC83855

[B7] Lacherade JC Cook D Heyland D Chrusch C Brochard L Brun-Buisson C Prevention of venous thromboembolism in critically ill medical patients: a Franco-Canadian cross-sectional study J Crit Care 2003 18 228 237 10.1016/j.jcrc.2003.10.006 14691896

[B8] Cook D Laporta D Skrobik Y Peters S Sharpe M Murphy P Chin D Crowther M Canadian ICU Directors Group Prevention of venous thromboembolism in critically ill surgery patients: a cross-sectional study J Crit Care 2001 16 161 166 10.1053/jcrc.2001.30665 11815901

[B9] Foster D Smith O Clarke F Thompson P Irwin M Hand L Laberge A O’Brien J Zytaruk N Kutsogiannis J Cook D Source data verification and calibration for critical care audits Crit Care Med 2012 40 A148

[B10] Knaus WA Draper EA Wagner DP Zimmerman JE APACHE II: a severity of disease classification system Crit Care Med 1985 13 818 829 10.1097/00003246-198510000-00009 3928249

[B11] Arnold DM Donahoe L Clarke FJ Tkaczyk AJ Heels-Ansdell D Zytaruk N Cook R Webert KE McDonald E Cook DJ Bleeding during critical illness: a prospective cohort study using a new measurement tool Clin Invest Med 2007 30 E93 E102 17716547 10.25011/cim.v30i2.985

[B12] Cook D Meade M Guyatt G Walter S Heels-Ansdell D Warkentin TE Zytaruk N Crowther M Geerts W Cooper DJ Vallance S Qushmaq I Rocha M Berwanger O Vlahakis NE PROTECT Investigators for the Canadian Critical Care Trials Group and the Australian and New Zealand Intensive Care Society Clinical Trials Group Dalteparin versus unfractionated heparin in critically ill patients N Engl J Med 2011 364 1305 1314 21417952 10.1056/NEJMoa1014475

[B13] Warkentin TE Sheppard JA Testing for heparin-induced thrombocytopenia antibodies Transfus Med Rev 2006 20 259 272 10.1016/j.tmrv.2006.05.001 17008164

[B14] Geerts WH Bergqvist D Pineo GF Heit JA Samama CM Lassen MR Colwell CW Prevention of venous thromboembolism: american college of chest physicians evidence-based clinical practice guidelines (8th edition) Chest 2008 133 381S 453S 10.1378/chest.08-0656 18574271

[B15] Alhazzani W Lim W Jaeschke R Murad H Cade J,DC Heparin thromboprophylaxis in medical-surgical ICU patients: a meta-analysis Crit Care Med 2013 41 2088 2098 10.1097/CCM.0b013e31828cf104 23782973

[B16] Lentine KL Flavin KE Gould MK Variability in the use of thromboprophylaxis and outcomes in critically ill medical patients Am J Med 2005 118 1373 1380 10.1016/j.amjmed.2004.12.025 16378781

[B17] Sinuff T Muscedere J Cook DJ Dodek PM Anderson W Keenan SP Wood G Tan R Haupt MT Miletin M Bouali R Jiang X Day AG Overvelde J Heyland DK Canadian Critical Care Trials Group Implementation of clinical practice guidelines for ventilator-associated pneumonia: a multicenter prospective study Crit Care Med 2013 41 15 23 10.1097/CCM.0b013e318265e874 23222254

[B18] Cook D Douketis J Meade M Guyatt G Zytaruk N Granton J Skrobik Y Albert M Fowler R Hébert P Pagliarello G Friedrich J Freitag A Karachi T Rabbat C Heels-Ansdell D Geerts W Crowther M Canadian Critical Care Trials Group Venous thromboembolism and bleeding in critically ill patients with severe renal insufficiency receiving dalteparin thromboprophylaxis: prevalence, incidence and risk factors Crit Care 2008 12 R32 10.1186/cc6810 18315876 PMC2447552

[B19] Lamontagne F Heels-Ansdell D McIntyre L Dodek P Meade M Skrobik Y McDonald E Vallance S Zytaruk N Cook D PROTECT Investigators on behalf of the CCCTG and the ANZICS-CTG Non-Leg venous thrombi during critical illness: a nested prospective cohort study Crit Care Med 2012 40 A758

[B20] Cook D Crowther M Meade M Rabbat C Griffith L Schiff D Geerts W Guyatt G Deep venous thrombosis in medical-surgical critically ill patients: prevalence, incidence, and risk factors Crit Care Med 2005 33 1565 1571 10.1097/01.CCM.0000171207.95319.B2 16003063

[B21] Shorr AF Williams MD Venous thromboembolism in critically ill patients. Observations from a randomized trial in sepsis Thromb Haemost 2009 101 139 144 19132200

[B22] Zarychanski R Lauzier F Turgeon A Marshall J Nates J Cox M Arabi Y Qushmaq I Zytaruk N Cook D VTE risk factors in medical-surgical ICU patients Crit Care Med 2011 39 A672

[B23] Zarychanski R Lim W Rocha M McIntyre L Lamontagne F Dodek P Pai M Cooper D Alhashemi J Zytaruk N Do statins influence DVT risk in critically Ill patients? Crit Care Med 2011 39 A112 10.1097/CCM.0b013e3181feb824

[B24] Zytaruk N Meade M Mehta S Hall R Granton J Ferguson N Freitag A Muscedere J Fowler R Skrobik Y Jacka M Marshall J Lauzier F Lamontagne F Turgeon A Vlahakis N Klinger J Devereaux PJ Ostermann M Berwanger O Khwaja K Burry L Freidrich J Burns K Herridge M Cooper D Bersten A Heels-Ansdell D Cook D Risk factors for pulmonary embolism in medical–surgical ICU patients Am J Resp Crit Care Med 2012 185 A2359

[B25] Mayr AJ Dunser M Jochberger S Fries D Klingler A Joannidis M Hasibeder W Schobersberger W Antifactor Xa activity in intensive care patients receiving thromboembolic prophylaxis with standard doses of enoxaparin Thromb Res 2002 105 201 204 10.1016/S0049-3848(02)00028-2 11927124

[B26] Robinson S Zincuk A Larsen UL Ekstrom C Nybo M Rasmussen B Toft P A comparative study of varying doses of enoxaparin for thromboprophylaxis in critically ill patients: a double-blinded, randomised controlled trial Crit Care 2013 17 R75 10.1186/cc12684 23601744 PMC4057520

[B27] Robinson S Zincuk A Strom T Larsen TB Rasmussen B Toft P Enoxaparin, effective dosage for intensive care patients: double-blinded, randomised clinical trial Crit Care 2010 14 R41 10.1186/cc8924 20298591 PMC2887151

[B28] Cook D Attia J Weaver B McDonald E Meade M Crowther M Venous thromboembolic disease: an observational study in medical–surgical intensive care unit patients J Crit Care 2000 15 127 132 10.1053/jcrc.2000.19224 11138871

[B29] Muscedere JG Heyland DK Cook D Venous thromboembolism in critical illness in a community intensive care unit J Crit Care 2007 22 285 289 10.1016/j.jcrc.2007.02.003 18086398

[B30] Schaden E Metnitz PG Pfanner G Heil S Pernerstorfer T Perger P Schoechl H Fries D Guetl M Kozek-Langenecker S Coagulation Day 2010: an Austrian survey on the routine of thromboprophylaxis in intensive care Intensive Care Med 2012 38 984 990 10.1007/s00134-012-2533-0 22446990

[B31] Dellinger RP Levy MM Rhodes A Annane D Gerlach H Opal SM Sevransky JE Sprung CL Douglas IS Jaeschke R Osborn TM Nunnally ME Townsend SR Reinhart K Kleinpell RM Angus DC Deutschman CS Machado FR Rubenfeld GD Webb S Beale RJ Vincent JL Moreno R Surviving Sepsis Campaign Guidelines Committee including The Pediatric Subgroup Surviving Sepsis Campaign: international guidelines for management of severe sepsis and septic shock, 2012 Intensive Care Med 2013 39 165 228 10.1007/s00134-012-2769-8 23361625 PMC7095153

[B32] Dorffler-Melly J de Jonge E Pont AC Meijers J Vroom MB Buller HR Levi M Bioavailability of subcutaneous low-molecular-weight heparin to patients on vasopressors Lancet 2002 359 849 850 10.1016/S0140-6736(02)07920-5 11897286

[B33] Priglinger U Delle Karth G Geppert A Joukhadar C Graf S Berger R Hulsmann M Spitzauer S Pabinger I Heinz G Prophylactic anticoagulation with enoxaparin: is the subcutaneous route appropriate in the critically ill? Crit Care Med 2003 31 1405 1409 10.1097/01.CCM.0000059725.60509.A0 12771610

[B34] Rommers MK Van der Lely N Egberts TC van den Bemt PM Anti-Xa activity after subcutaneous administration of dalteparin in ICU patients with and without subcutaneous oedema: a pilot study Crit Care 2006 10 R93 10.1186/cc4952 16790078 PMC1550968

[B35] Gould MK Garcia DA Wren SM Karanicolas PJ Arcelus JI Heit JA Samama CM Prevention of VTE in nonorthopedic surgical patients: antithrombotic therapy and prevention of thrombosis, 9th ed: American College of Chest Physicians evidence-based clinical practice guidelines Chest 2012 141 e227S e277S 22315263 10.1378/chest.11-2297 PMC3278061

[B36] Douketis J Cook D Meade M Guyatt G Geerts W Skrobik Y Albert M Granton J Hébert P Pagliarello G Marshall J Fowler R Freitag A Rabbat C Anderson D Zytaruk N Heels-Ansdell D Crowther M Canadian Critical Care Trials Group Prophylaxis against deep vein thrombosis in critically ill patients with severe renal insufficiency with the low-molecular-weight heparin dalteparin: an assessment of safety and pharmacodynamics: the DIRECT study Arch Intern Med 2008 168 1805 1812 10.1001/archinte.168.16.1805 18779469

[B37] Mahe I Aghassarian M Drouet L Bal Dit-Sollier C Lacut K Heilmann JJ Mottier D Bergmann JF Tinzaparin and enoxaparin given at prophylactic dose for eight days in medical elderly patients with impaired renal function: a comparative pharmacokinetic study Thromb Haemost 2007 97 581 586 17393021

[B38] Limpus A Chaboyer W McDonald E Thalib L Mechanical thromboprophylaxis in critically ill patients: a systematic review and meta-analysis Am J Crit Care 2006 15 402 410 quiz/discussion 411–412 16823018

[B39] PREPIC Study Group Eight-year follow-up of patients with permanent vena cava filters in the prevention of pulmonary embolism: the PREPIC (Prevention du Risque d’Embolie Pulmonaire par Interruption Cave) randomized study Circulation 2005 112 416 422 16009794 10.1161/CIRCULATIONAHA.104.512834

[B40] Sarosiek S Crowther M Sloan JM Indications, complications, and management of inferior vena cava filters: the experience in 952 patients at an academic hospital with a level I trauma center JAMA Intern Med 2013 173 513 517 10.1001/jamainternmed.2013.343 23552968

[B41] Tapson VF Decousus H Pini M Chong BH Froehlich JB Monreal M Spyropoulos AC Merli GJ Zotz RB Bergmann JF Pavanello R Turpie AG Nakamura M Piovella F Kakkar AK Spencer FA Fitzgerald G Anderson FA Jr IMPROVE Investigators Venous thromboembolism prophylaxis in acutely ill hospitalized medical patients: findings from the International Medical Prevention Registry on Venous Thromboembolism Chest 2007 132 936 945 10.1378/chest.06-2993 17573514

[B42] Cook D Tkaczyk A Lutz K McMullin J Haynes RB Douketis J Thromboprophylaxis for hospitalized medical patients: a multicenter qualitative study J Hosp Med 2009 4 269 275 10.1002/jhm.461 19504488

[B43] McMullin J Cook D Griffith L McDonald E Clarke F Guyatt G Gibson J Crowther M Minimizing errors of omission: behavioural reenforcement of heparin to avert venous emboli: the BEHAVE study Crit Care Med 2006 34 694 699 10.1097/01.CCM.0000201886.84135.CB 16505655

[B44] Johnston G Crombie IK Davies HT Alder EM Millard A Reviewing audit: barriers and facilitating factors for effective clinical audit Qual Health Care 2000 9 23 36 10848367 10.1136/qhc.9.1.23 PMC1743496

[B45] Scott IA Harper CM Guideline-discordant care in acute myocardial infarction: predictors and outcomes Med J Aust 2002 177 26 31 12088475 10.5694/j.1326-5377.2002.tb04627.x

[B46] Vittinghoff E McCulloch CE Relaxing the rule of ten events per variable in logistic and Cox regression Am J Epidemiol 2007 165 710 718 10.1093/aje/kwk052 17182981

